# Activity of semicarbazide-sensitive amine oxidase in guinea-pig tissues is not affected by metformin

**DOI:** 10.1186/2050-6511-13-S1-A79

**Published:** 2012-09-17

**Authors:** Katja Lukan, Simona Rajtar Osredkar

**Affiliations:** 1 Institute for Pharmacology and Experimental Toxicology, Faculty of Medicine, University of Ljubljana, 1000 Ljubljana, Slovenia

## Background

Semicarbazide-sensitive amine oxidase (SSAO) is an enzyme that performs oxidative deamination of primary amines to aldehyde, ammonia and hydrogen peroxide. It appears that these products may have important signalling function in the regulation of cell development and glucose homeostasis. Much attention has been given to raised plasma SSAO activity in diabetic patients, both types 1 and 2. We were interested whether the peroral antidiabetic drug metformin affects SSAO activity *in vitro*. In this study we used metformin because it is usually the first-line medication used for treatment of type 2 diabetes.

## Methods

First we studied the activity of SSAO in 15 different guinea-pig tissues with radiochemical methods. Two of 15 tissues (liver and kidney) were also used for the *in vitro* experiment, in which preincubation with metformin or the SSAO inhibitor semicarbazide was performed. Tissue homogenates were used for protein quantification using the Bradford method and for determination of SSAO activity using radioactive [^14^C]benzylamine as substrate. Since benzylamine is also a substrate for MAO-B, the MAO-B inhibitor deprenyl was added to the incubation mix. After 30 min of incubation at 37°C in sodium phosphate buffer the reaction was stopped by addition of perchloric acid. The product [^14^C]benzaldehyde was extracted into toluene containing 0.6% 2,5-diphenyloxazol. We measured radioactivity in the organic phase with a liquid scintillation counter.

## Results

The highest concentration of SSAO was determined in the gastrointestinal system, especially in the liver (32 ± 4.0 mU/mg protein). Relatively high SSAO activity was found also in the spleen (47 ± 4.8 mU/mg protein), abdominal artery (39 ± 8.5 mU/mg protein), skin (32 ± 9.0 mU/mg protein), kidney (33 ± 4.7 mU/mg protein) and lung (26 ± 2.6 mU/mg protein). Smaller amounts of SSAO were present in pancreas and brain. Our results indicated that metformin had no effect on SSAO activity *in vitro*. As expected, semicarbazide decreased SSAO activity in a concentration-dependent manner.

## Conclusions

SSAO appears to have an important role in the gastrointestinal system of the guinea-pig. The high SSAO concentration in the liver is probably due to the detoxifying function of this tissue. The relatively high SSAO levels in the lung could protect the animals from inhaled methylamine and other volatile amines. We conclude that SSAO activity is probably not affected during treatment with the antidiabetic drug metformin.

